# High Rate of Circulating MERS-CoV in Dromedary Camels at Slaughterhouses in Riyadh, 2019

**DOI:** 10.3390/v12111215

**Published:** 2020-10-27

**Authors:** Taibah A. Aljasim, Abdulrahman Almasoud, Haya A. Aljami, Mohamed W. Alenazi, Suliman A. Alsagaby, Asma N. Alsaleh, Naif Khalaf Alharbi

**Affiliations:** 1 Vaccine Development Unit, Department of Infectious Disease Research, King Abdullah International Medical Research Center, Riyadh 11564, Saudi Arabia; 438203825@student.ksu.edu.sa (T.A.A.); almasoudab@ngha.med.sa (A.A.); aljamiha@ngha.med.sa (H.A.A.); alenazimo8@ngha.med.sa (M.W.A.); 2 College of Science, King Saud University, Riyadh 11564, Saudi Arabia; asmalsaleh@ksu.edu.sa; 3 King Saud bin Abdulaziz University for Health Sciences, Riyadh 11564, Saudi Arabia; 4 Department of Medical Laboratories Sciences, College of Applied Medical Sciences, Majmaah University, Majmaah 11952, Saudi Arabia; s.alsaqaby@mu.edu.sa

**Keywords:** MERS-CoV, camel, seroprevalence, transmission, ELISA, RT-PCR, slaughterhouse

## Abstract

MERS-CoV is a zoonotic virus that has emerged in humans in 2012 and caused severe respiratory illness with a mortality rate of 34.4%. Since its appearance, MERS-CoV has been reported in 27 countries and most of these cases were in Saudi Arabia. So far, dromedaries are considered to be the intermediate host and the only known source of human infection. This study was designed to determine the seroprevalence and the infection rate of MERS-CoV in slaughtered food-camels in Riyadh, Saudi Arabia. A total of 171 nasal swabs along with 161 serum samples were collected during the winter; from January to April 2019. Nasal swabs were examined by Rapid test and RT-PCR to detect MERS-CoV RNA, while serum samples were tested primarily using S1-based ELISA Kit to detect MERS-CoV (IgG) antibodies and subsequently by MERS pseudotyped viral particles (MERSpp) neutralization assay for confirmation. Genetic diversity of the positive isolates was determined based on the amplification and sequencing of the spike gene. Our results showed high prevalence (38.6%) of MERS-CoV infection in slaughtered camels and high seropositivity (70.8%) during the time of the study. These data indicate previous and ongoing MERS-CoV infection in camels. Phylogenic analysis revealed relatively low genetic variability among our isolated samples. When these isolates were aligned against published spike sequences of MERS-CoV, deposited in global databases, there was sequence similarity of 94%. High seroprevalence and high genetic stability of MERS-CoV in camels indicating that camels pose a public health threat. The widespread MERS-CoV infections in camels might lead to a risk of future zoonotic transmission into people with direct contact with these infected camels. This study confirms re-infections in camels, highlighting a challenge for vaccine development when it comes to protective immunity.

## 1. Introduction

Middle East Respiratory Syndrome (MERS), caused by MERS Coronavirus (MERS-CoV) is one of three recently emerged human coronaviruses that cause severe respiratory infections and were associated with major outbreaks worldwide. Among the three emerged human coronaviruses, MERS-CoV is considered the most lethal with a mortality rate of approximately 34.4% of all cases [1]. The symptoms of the infection vary from asymptomatic or mild including; fever, cough, shortness of breath, and occasionally myalgia, sore throat and hemoptysis [2] to the rapid progression of acute pneumonia, respiratory destress or multi-organ failure and death [1]. Since its emergence, MERS-CoV has infected around 2900 people and caused 858 deaths in 27 countries. The majority of the cases were reported from Saudi Arabia (2102 cases; 72%), including 780 related deaths [1]. The virus was first detected in Saudi Arabia in 2012, from a patient with severe respiratory illness and subsequent multi-organ failure and death [3]. Molecular and sero-epidemiological data on MERS-CoV infections in humans indicated a zoonotic transmission from dromedary camels [4,5]. Camels were confirmed as intermediate animal host showing high proportions of MERS-CoV circulation especially in young calves (<2 years old) in many Asian and Middle Eastern countries including Saudi Arabia where 56.4% and 44.9% of the tested dromedaries were shedding MERS-CoV in two separate studies by 2015 [6,7]. Other studies reported viral detection in 59% of the calves presented for slaughter in Qatar, with high seropositivity of 97% [8] In Dubai, 73% of tested calves were MERS-CoV positive, with seropositivity of 93% of the overall test camels [9]. Low infection rate of 3.6% was reported in adult camels (>4 years old) in Egypt, with high seroprevalence 92% in all tested camels [10].

MERS-CoV enters mammalian cells via the dipeptidyl peptidase-4 (DPP4) that is expressed on the surface of animal cell lines derived from goats, sheep and cows; however, anti-MERS-CoV antibodies were only detected in camels, mainly in adult >2 years old, with high seroprevalence rate of (74% to 100%) in several Asian, African, and Middle Eastern countries including Saudi Arabia [4,8,9,11,12,13,14,15] Anti-MERS-CoV antibodies have been detected in camel sera archived 20 to 30 years ago in African countries [15], UAE [5], and Saudi Arabia [16]. However, pre-existing antibodies are not necessarily protective from repeated viral infections in camels and re-infection of camels could occur [17,18]. Camel-to-human transmission could occur during direct contact with infected camel nasal secretions, saliva, and respiratory droplets [19]. Almost 54% of primary human cases have reported direct or indirect camel contact [20] and although camel workers are not usually symptomatic and their viral infection is only detected retrospectively by serology testing, 50% of camel workers were found to be seropositive [21].

Despite public health measures, such as relocating camel markets to the outskirt of cities and strict hygiene and close monitoring of camel slaughterhouses and butcher shops, Saudi Arabia reports by far the most MERS cases. The country has a large population of dromedary camels, yet it is lower than that of Eastern Africa [22]. Although camels are the confirmed direct zoonotic source of the infection, several gaps in knowledge remain unaddressed, such as the mechanism of virus transmission from camels to humans, the low rate of symptomatic infection in camel workers, the potential infectiousness of asymptomatic cases, and the rate of virus circulation in camels every year. To get a better understanding of MERS-CoV zoonotic transmission and to prevent further outbreaks resulting from camel contact, continuous surveillance on the virus circulation, prevalence, and genetic changes in the viral genome are important and highly warranted, especially in epidemic countries such as Saudi Arabia. Here, this study aims to report recent serological and molecular data on MERS-CoV to provide recent data and to support establishing active surveillance in endemic countries. The study focuses on screening camels that are brought for food production in Riyadh slaughterhouses in Saudi Arabia, during the winter season from January to April of 2019. Riyadh was selected as a highly populated city as well as having a large population of camels [22]. Therefore, this study assesses the presence of MERS-CoV, the genetic sequences of the spike gene, and anti-MERS-CoV neutralizing antibodies in this population of dromedary camels at a single time point, prior to slaughtering, between January and April of 2019.

## 2. Materials and Methods

### 2.1. Samples

A total of 171 camels in the three main slaughterhouses in Riyadh city were sampled for blood and nasal swabs immediately after culling, during the winter of 2019 (January 2019 to April 2019). Nasal swabs were collected using rayon flexible swabs and placed in 3 mL of universal transport media (UTM) (COPAN Italia, Brescia, Italy). A second nasal swab was used for Rapid test. Blood samples were collected in sterile plain tubes, left to clot, and spun for serum isolation. All samples were aliquoted and kept in −80 °C freezer until the time of analysis. The age of camels was not documented but the majority of camels in slaughterhouses were young and healthy (for food production), estimated to be between 2 and 4 years.

### 2.2. Rapid MERS-CoV Ag Assay

All nasal swabs were tested using Rapid MERS-CoV Ag Test Kit (BioNote Inc., Hwaseong, South Korea), referred to as Rapid test, which is an immunochromatographic assay used for qualitative detection of MERS-CoV on the spot after sample collection. Following the manufacturer’s instruction, nasal swabs were transferred directly into a test tube containing the assay diluents. Then, the test strip was placed inside that tube. Results were read within 15 min ensuring the control (C) line appears. Samples were considered positive when both the test lines (T) and C appeared, and negative if only the C appeared. In the absence of C, the test was considered invalid and was repeated.

### 2.3. Reverse Transcriptase Polymerase Chain Reaction (RT-PCR)

RT-PCR was performed following standard procedure. In details, MERS-CoV RNA were extracted from 140 μL of each nasal swab’s VTM by QIAamp viral RNA mini kit (Qiagen, Hilden, Germany) following the manufacturer’s protocol. Extracted RNA was used to synthesize cDNA using High Capacity cDNA Reverse transcription kit (Thermo Fisher Scientific, Waltham, MA, USA) according to the manufacturer’s protocol. For MERS-CoV screening, open reading frame ORF1a gene region was targeted by the following oligos: Forward primer (CCACTACTCCCATTTCGTCAG), Reverse primer (TGCTTATATGCGCACTACACATACTG), and Taq Man probe [FAM]-TTGCAAATTGGCTTGCCCCCACT-[TAMRA]. FAM is the fluorescent reporter dye that is 6-carboxyfluorescein while TAMRA is the fluorescent quencher. A 20 µL reaction was set up containing: 2 µL of cDNA, 10 µL of TaqMan Gene Expression Master Mix (2×), 1 µL of 5 µM probe (0.5 µM) 6-carboxyfluorescein, 1 µL of 5 µM of both Primers (0.5 µM each) and 6 µL of deionized water. All samples were run in duplicate in MicroAmp™ Optical 96-Well Reaction Plate and analyzed using RQ Manager version1.2.1 (Applied Biosystems, Foster City, CA, USA) using Applied Biosystems 7900 HT Fast Real-time PCR System. The reaction conditions were as follow: 50 °C for 2 min, 95 °C for 10 min, and 40 cycles of 95 °C for 15 s and 60 °C for 1 min. Samples that had been collected from healthy naïve calves and confirmed negative in a previous study were used as negative controls in this assay. The positive cutoff was determined as the lowest Ct value of the negative controls plus 3 SD of the average of negative controls.

### 2.4. ELISA

Serum samples were evaluated using S1-based ELISA Kit for detecting IgG antibodies (EUROIMMUN, Lübeck, Germany). According to the manufacturer’s instructions. The sample ratio over the kit calibrator was reported as the main readout; ratios >1.1 and (0.8 to 1.1) are considered positive or equivocal, respectively.

### 2.5. MERS Pseudotyped Viral Particles (MERSpp) Neutralization Assay

MERS pseudotyped viral particles (MERSpp) were produced and titrated using Huh7.5 cells as described previously [23,24]. Camel serum samples were heat-inactivated and prepared in a 3-fold serial dilution starting from 1:20 and tested for neutralizing antibodies in duplicate A standard concentration of MERSpp (equivalent to 200,000 Relative Luminescence Units (RLU) and Huh7.5 cells (10,000 cells) were added to each well. Cells only and cells with MERSpp only (both without serum) were included in quadruplicate as controls to determine 100% and 0% neutralization activity, respectively. Following 48 h incubation, cells were lysed, and the assay was developed using Bright-Glo™ Luciferase Assay System (Promega Madison, WIS, USA) and luciferase activity was measured using a luminometer. Thus, 50% of inhibitory concentration (IC50) neutralization titers (in Log^10^) were calculated for each serum sample using GraphPad Prism [25].

### 2.6. Sequencing and Phylogenic Analysis

The spike gene was amplified by eight pairs of primers (Table S1) spanning the full-length spike from cDNA made from viral RNA, extracted from nasal swabs. The PCR was performed using DreamTaq™ Hot Start Green PCR Master Mix with 25 µL reaction containing: 2 µL of cDNA, 12.5 µL of Master Mix, 1 µL of 5 µM of each primer and 8.5 of nuclease free water. The thermo cycle was set up in sequential steps as 98 °C for 1 min, and 30 cycles of 98 °C for 10 s, 62–63 °C for 20 s and 72 °C for 40 s, and final extension at 72 °C for 10 min. Each of the PCR product fragments range from 486 to 802 bp in size. Sanger sequencing was preformed using BigDye^®^ Terminator v3.1 Cycle Sequencing Kit (Applied Biosystems, Foster City, CA, USA). Briefly, 5 µL of PCR products were enzymatically purified using 2 µL of ExoSAP-IT™ (Thermo Fisher Scientific, Waltham, MA, USA) and placed in the thermal cycler for 30 min at 37 °C flowed by 15 min at 80 °C. Spike gene sequencing was performed in a 10 µL reaction containing: 2 µL of BigDye™ Terminator 3.1 Ready Reaction Mix, 1 µL of BigDye™ Terminator v1.1 & v3.1 5× Sequencing Buffer, 1 µL of 5 µM of either forward or reverse primer, 2 µL of DNA template (ranging from 5 to 20 ng) and 4 µL of deionized water. The cycling conditions as follows: 96 °C for 1 min, 30 cycles consisting of 96 °C for 10 s, 50 °C for 5 s and 60 °C for 4 min. The resulted PCR products of the sequencing were purified using 45 µL of SAM™ solution and 10 µL of X Terminator™ Solution (applied biosystems, USA) for each sample, placed in the plate vortex for 1 h at 1900 and run in the 3730xL DNA Analyzer Sanger sequencer (Applied Biosystems, Foster City, CA, USA). The product sequences were aligned against 250 MERS-CoV Spike gene sequences (from camel isolates) that were obtained from GenBank database (GenBank, NCBI, 2020), deposited between 2012 and 2017, using MUSCLE alignment method in Geneious prime (version 2020.1.3). To compare between the sequence of the isolates, the distance matrix was calculated by Euclidean measurement method and a pairwise heatmap was built using heatmapper [26].

### 2.7. Ethical Approval

This study was approved by IRB and IACUC at KAIMRC for the protocol number: RC17/220.

### 2.8. Statistical Analysis

Data were collected from ELISA reader as OD values, from Neutralization assay plate reader as OD values, and from PCR machine as Ct values. The data values were plotted and analyzed using GraphPad Prism software (GraphPad Software Inc., San Diego, CA, USA).

## 3. Results

### 3.1. Detection of MERS-CoV in Slaughterhouse Camels in Riyadh

**Figure 1:**
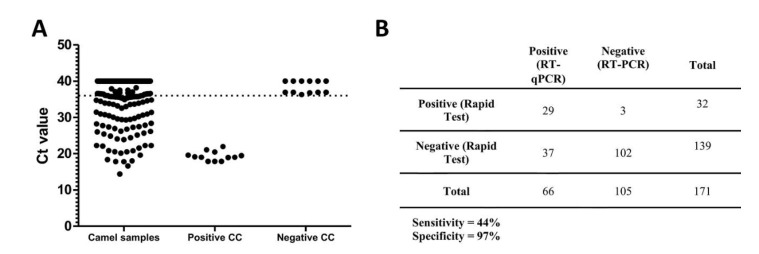
Molecular detection of MERS-CoV in slaughterhouse camels in Riyadh, 2019. (**A**): Camel nasal swab samples tested by RT-PCR (*n* = 171) and presented as Ct values. Negative control and positive control samples were tested in this assay. Samples are considered positive if their values is below the dotted line, which represent the cutoff of the assay. (**B**): A comparison between the RT-PCR and the Rapid test representing the sensitivity and specificity of the Rapid test.

In order to determine the rate of MERS-CoV in camels slaughtered for food in Riyadh during the winter of 2019, 171 camels were sampled from three large official abattoirs in Riyadh city. RT-PCR was used to screen the samples in duplicate and showed that 66 samples were positive (below the cutoff Ct value), with an infection rate of 38.6% (Figure 1A,B). The Ct values of all tested samples ranged from 14.377 to 40. This finding indicates an ongoing circulation of MERS-CoV in camels in Saudi Arabia, as tested in camels brought from different locations into slaughterhouses for food production. Camel nasal swabs were also examined using the Rapid test for MERS-CoV antigen at site during the sampling. The prevalence of MERS infection among slaughtered camels during the time of collection was 18.7%, 32 positive samples, by the Rapid test. As compared to 66 positive samples by RT-PCR, the Rapid test detect only 29 of these samples, showing a low sensitivity of 44%, Figure 1B.

### 3.2. Genetic Sequence of MERS-CoV Circulating in Slaughterhouse Camels in Riyadh

**Figure 2:**
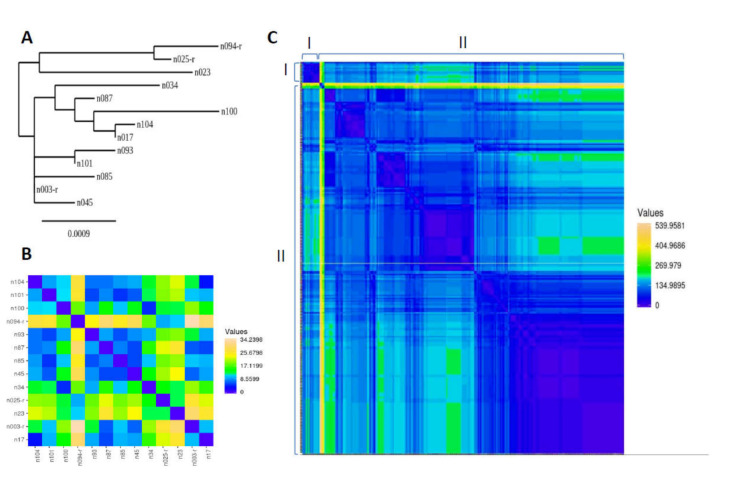
Genetic analysis of spike gene sequences isolated from slaughterhouse camels in Riyadh, 2019. (**A**): Phylogenetic tree based on nucleotide sequences of spike gene of the 13 isolates of this study. (**B**): Pairwise heatmap presentation of the 13 sequences (**C**): Heatmap to compare between MERS-CoV spike gene sequences obtained from (I) the current study (*n* = 13) and (II) archived sequences obtained from GenBank (*n* = 250). The color scale is shown to indicate the distance value between sequences.

To assess the genetic variability of the spike gene among selected positive samples, eight primers were designed to amplify overlapping segments of the spike gene. Subsequently, eighteen RT-PCR positive isolates were selected based on their Ct value of lower than 25 and used for spike gene amplification. Sanger sequencing was preformed to reveal the spike gene sequences in these samples. Of which, only 13 samples were successfully sequenced and assembled; the assembled sequences were aligned to the EMC/2012 MERS-CoV reference sequence (Genbank Ref: NC_019843.3). The 13 sequences showed high degree of similarity to each other (identical positions = 4025 nucleotides out of 4062; homology = 98.99%). The phylogenetic tree analysis and stepwise heatmap (Figure 2A,B) showed that the 13 sequences were allocated in two main clusters; the sequences n094-r, n025-4 and n023 in one cluster and the remaining 10 sequences in another cluster. The most similar sequences were n104 and n017 that were closely clustered together with a distance value of 3.60. The sequences n094-r and n003-r are examples of remotely clustered sequences among the 13 isolates with a distance value of 34.24. Agreeing with the phylogenetic finding (Figure 2A), the pairwise heatmap (Figure 2B) showed that the sequence n94-r recorded high distance values with the majority of the other isolate sequences (distance value of 9.41 to 34.24; median = 25.78), highlighting that n094-r is the most different sequence among the 13 isolate sequences. The alignment of the 13 sequences to the reference sequence is also shown in a heatmap, Figure S1. In support of the similarity analyses above, it is important to note that samples from the infected camels were collected on different days during the study duration of four months. Samples were collected at three different slaughterhouses, which receive individual camels from various small camel barns, farms, and markets that have different locations across the province of Riyadh, beyond Riyadh city. Therefore, it is unlikely that these 13 isolates are coming from the same location or at the same time of collection.

Alignment of our sequences against previously published sequences (*n* = 250) of MERS-CoV isolated from camels showed 94.0% similarity (identical locations were 3822 out of 4066 bp) as supported by the stepwise heatmap, Figure 2C. The least similarity was observed when our 13 samples were aligned to the MERS-CoV sequence Ref: KX108943.1, which was a MERS-CoV camel isolate from UAE in 2016.

### 3.3. Seroprevalence of MERS-CoV in Slaughterhouse Camels in Riyadh

**Figure 3:**
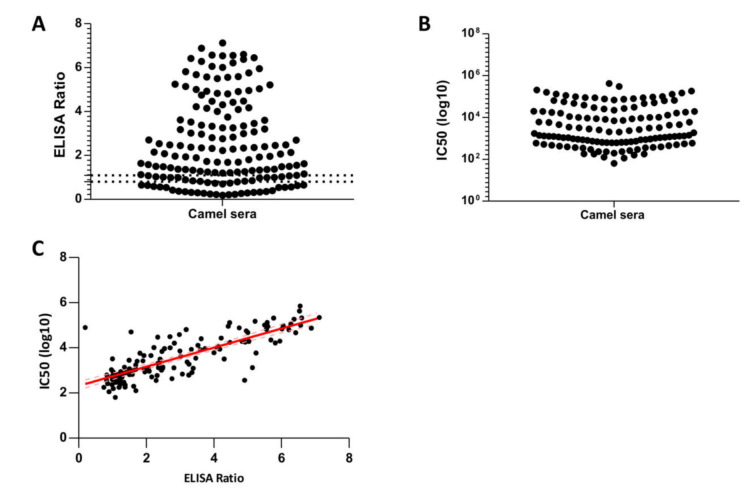
Seroprevalence of MERS-CoV in slaughterhouse camels in Riyadh, 2019. (**A**): Anti-MERS-CoV antibody levels in the tested camel sera using S1-based ELISA Kit. Samples are considered positive when ELISA ratio is above 1.1. (**B**): Neutralizing anti-MERS-CoV antibodies in camel sera were confirmed in all samples that are positive by ELISA. This is shown as 50% inhibitory concentration of MERS pseudotyped viral particles in log scale. (**C**): Correlation between ELISA ratios and titres of neutralizing antibodies by MERSpp assay. Pearson correlation coefficient of 0.80, R squared of 0.65, and a *p*-value of <0.0001.

In order to estimate the prevalence of MERS-CoV neutralizing antibodies in the tested camels, 161 serum samples were collected from the 171 camels that were samples for nasal swabs. Using S1-based commercial ELISA, serum samples showed evidence of MERS-CoV antibodies in 114 camels, indicating a seropositivity rate of 70.8%. Thirty camels were seronegative and 16 showed equivocal antibody response in the ELISA, Figure 3A. Notably, among these 114 camels there were 37 positive by qRT-PCR. This shows that 37 out of the 66 infected camels (as confirmed by RT-PCR) had detectable antibody responses, highlighting MERS re-infection in camels. To confirm this data further, the serum samples, positive by ELISA, were assayed in a subsequent MERS pseudotyped viral particles (MERSpp) neutralization assay. Neutralization activity of the were confirmed in all ELISA positive sera, with variable titers, ranging from IC50 (${\mathrm{Log}}^{10}$) of 102 to 106, Figure 3B. The ELISA and MERSpp neutralization assay were shown to be in a good correlation, with Pearson correlation coefficient of 0.80, R squared of 0.65, and a *p*-value of <0.0001, Figure 3C. These findings indicate high seroprevalence in camels in Saudi Arabia with evidence of re-infection, therefore, these antibody responses might be a result of previous single or multiple MERS-CoV infections.

## 4. Discussion

Dromedary camels are so far the only confirmed animal source of Middle East Respiratory Syndrome coronavirus (MERS-CoV) infection in humans. However, the exact route of zoonotic transmission is yet to be confirmed. In this study we evaluated the rate of MERS-CoV infection as well as the prevalence of MERS-CoV antibodies in dromedaries at three slaughterhouses in Riyadh city in the Winter of 2019 (January to April). This study also assessed the genetic variability in the spike gene among the PCR strongly positive isolates with low Ct values ranging from 14 to 25. This study shows high rates of MERS-CoV infection as well as high rate of MERS-CoV neutralizing antibodies among dromedary camels in slaughterhouses in Riyadh. The overall prevalence of MERS infection in camels by RT-PCR was 38.6%. Previous studies reported higher prevalence of MERS-CoV in camels including a prevalence of 56.4% in dromedaries in Saudi Arabia, 2015 to 2017 [27], and 59% of slaughterhouse camels in Qatar, 2014 [28]. Although this may indicate higher circulation of MERS-CoV in camels in Saudi Arabia, this high rate could be considered as a reflection of MERS-CoV prevalence in camels in Eastern Africa; vast majority of camels in Saudi Arabia are imported on a continuous basis from Somalia, Djibouti, Sudan, and Ethiopia [29]. Our results indicate an ongoing infection of MERS in camels despite almost a decade since its first identification in camels in Saudi Arabia; this virus still circulates in camels in major cities of the country. However, it is worth noting that the slaughterhouses visited in these studies have strict public health measures, cleanliness, and proper hygiene. Most of the slaughtering process and meat preparation are done with semi-automation handling of the animals and in areas with restricted authorized access; yet, this high rate of viral infection in camels is worrisome.

The study showed an overall high seropositivity of MERS-CoV in camel serum samples (70.8%), which is consistent with previous studies that reported high prevalence of MERS-CoV antibodies in camels, ranging from 74% to 100%, in multiple African and Arabian countries [5,7,9,12,13,14,15,16,17,18]. Some infected camels in the current study seemed to have pre-existing MERS-CoV antibodies; this is supported by our previous data that showed MERS-CoV viral RNA is detectable for 14 days post infection in seronegative camels and that antibody responses in these camels would take 28 days post infection to be detectable [25], in addition to other studies that highlighted MERS-CoV re-infection in camels [17,22,25]. Some of which studied MERS-CoV seroprevalence in young camels; showing that MERS-CoV antibodies were found in >96% of calves above 2 years of age and 80% of calves below 1 year of age in Dubai, UAE [9]. Our results from the commercial anti-MERS-CoV specific ELISA showed high level of antibody ratios (indirect measurement of antibody titres) that were subsequently confirmed by an in-house (MERSpp) neutralization assay. However, it seems that MERS-CoV neutralizing antibodies in camels do not prevent against re-infection. High prevalence of MERS-CoV and MERS-CoV antibodies indicates ongoing infection among camels that are in contact with humans, which urges the need for more precautions and public health measures to prevent further zoonotic transmission and outbreaks; especially for people in close contact with these camels including abattoir workers, veterinary doctors and camel workers.

Genetic diversity has been reported in some studies particularly in the spike gene, which has a main role in tropism and transmissibility. Such studies reported high genetic variability with other published MERS-CoV sequences, the nucleotide sequence identity ranged from 65.7% to 99.8% [29]. However, other studies reported relatively high genetic stability among MERS-CoV isolates, which is consistent with our findings [8,30]. MERS-CoV spike sequences from our study were assembled to 250 MERS-CoV spike sequences (collected from camel isolates between 2012 to 2017 and deposited in the GenBank). Data analysis showed considerable degree of a conserved spike, the overall similarity was 94% to previously published sequences of MERS-CoV from camel isolates. Moreover, despite being collected at different times from different abattoirs, alignment of our sequences revealed high genetic similarity (homology = 98.99%) and the phylogenetic analysis revealed that all the 13 sequences are clustering together when aligned against other MERS-CoV sequences. Our isolates were found to be genetically closer to sequences collected from Saudi Arabia rather than other isolates from other gulf countries like UAE. The relatively low genetic diversity among MERS-CoV isolates (in the spike gene) since its emergence in 2012 combined with the high prevalence of MERS-CoV virus in camels and the presence of MERS-CoV antibodies in camels without preventing new infections are all indicating that it might be hypothesized that camels may not just be the intermediate host but might be considered as a natural host of MERS-CoV [17]; and this hypothesis would need further experimental evidence. Continuous surveillance of MERS-CoV infections in camels and further sequencing of the viral genome can improve our understanding and prediction of MERS-CoV evolution pattern.

Overall, this study presents a recent data on the ongoing circulation of MERS-CoV in dromedary in the biggest city of Saudi Arabia, which is one of the most MERS-CoV endemic countries. Since MERS is a zoonotic disease that can cause epidemics and outbreaks in humans, more studies would be needed to evaluate the impact of this current status of MERS-CoV in camels on public health and infection control. The study also supports vaccine development based on the spike gene as well as highlighting the challenge of re-infection in camels. The latter is an issue for vaccine development when establishing a protective titre or correlate of protection in camels.

## Supplementary Materials

The following are available online at https://www.mdpi.com/1999-4915/12/11/1215/s1, Figure S1: Genetic analysis of spike gene sequences isolated from slaughterhouse camels in Riyadh, 2019, Table S1: Primers used for amplification of the MERS-CoV spike gene.
