# Supplementary material for: High Rate of Circulating MERS-CoV in Dromedary Camels at Slaughterhouses in Riyadh, 2019
Source: Viruses. 2020 Oct 27;12(11):1215. doi: 10.3390/v12111215 (PMC7692456; doi:10.3390/v12111215)
Supplement: Supplementary file 1 [file viruses-12-01215-s001.pdf]

Supplementary file

Table 1. Primers used for amplification of the MERS-CoV spike gene.

No.	Primer Name	Primer Sequence
1	Seq-spk-F1	CTCTTGGTGGGTCTGTTGCTA
2	Seq-spk-R1	TTTTGTGGAGTTGTGCCTGT
3	Seq-spk-F2	TTTCCCTATCAGGGAGACCA
4	Seq-spk-R2	ATACGTAGAAGGCAGCCCAA
5	Seq-spk-F3	TTGTACGGCGGCAATATGT
6	Seq-spk-R3	AATCACCGTCTTCCCACACA
7	Seq-spk-F4	TGCTCTCGTCTTCTTTCTGATG
8	Seq-spk-R4	ACTTGCAGTCCTCTACGAACA
9	Seq-spk-F5	CGGCGAGATTCTACATATGGC
10	Seq-spk-R5	CTGATGCTGGACCTTGCTG
11	Seq-spk-F6	GGCAGTCGTAGTGCACGTAGT
12	Seq-spk-R6	CTGCTGTGCAACAAAAGCAT
13	Seq-spk-F7	CGCCTCTATTGGAGACATCA
14	Seq-spk-R7	GTACCACGGCCATTTGTTGT
15	Seq-spk-F8	CCTACGAGATGTTGTCTCTTCAA
16	Seq-spk-R8	TCTGCAGATGGGACGTCAAT

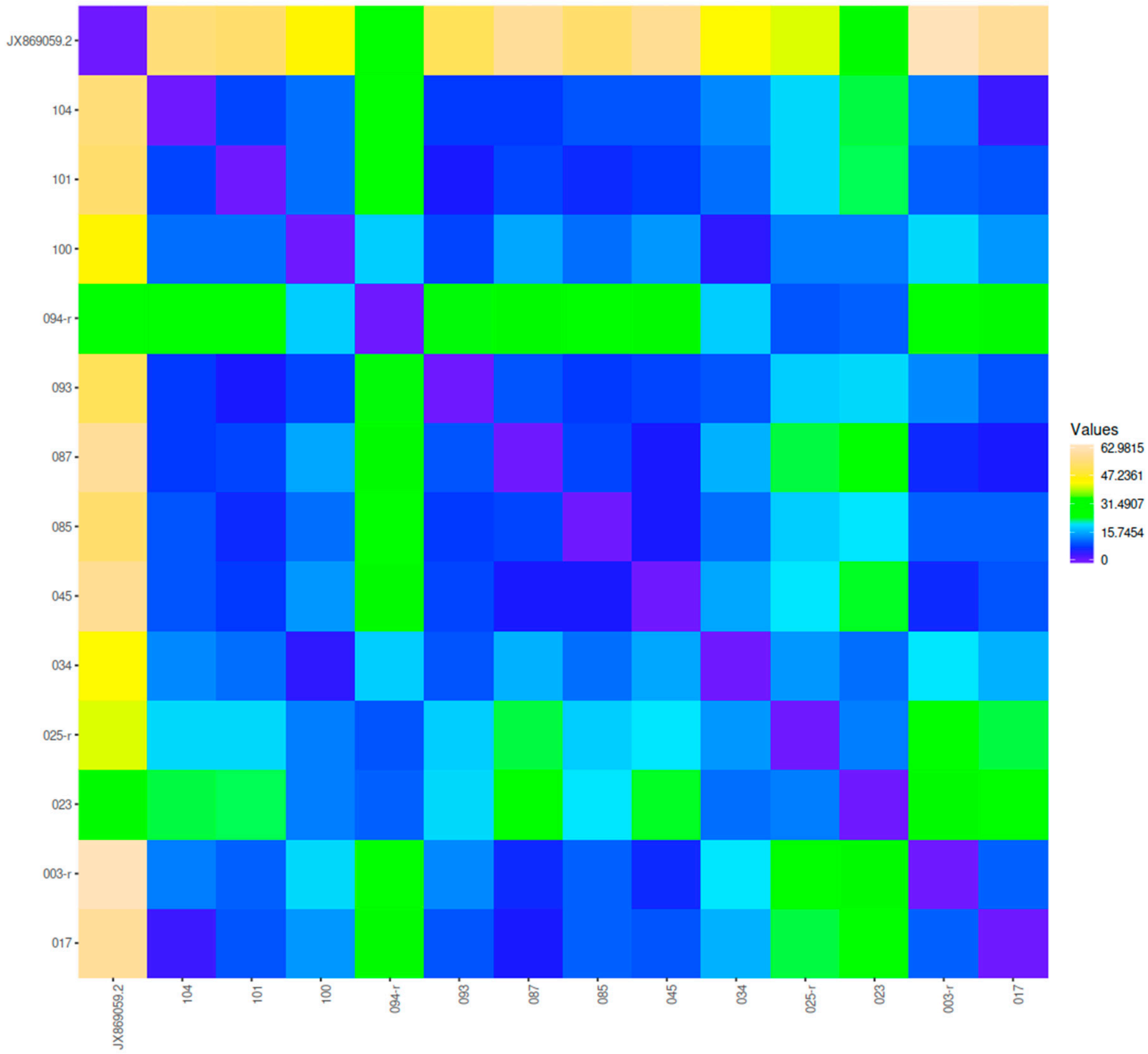

Figure 1. Genetic analysis of spike gene sequences isolated from slaughterhouse camels in Riyadh, 2019. Pairwise heatmap presentation of the 13 sequences obtained from the current study as compared to a MERS-CoV reference genome (Genbank Ref: NC_019843.3).
